# Differential effect of morphine on gastrointestinal transit, colonic contractions and nerve-evoked relaxations in Toll-Like Receptor deficient mice

**DOI:** 10.1038/s41598-018-23717-4

**Published:** 2018-04-12

**Authors:** Elizabeth A. H. Beckett, Vasiliki Staikopoulos, Mark R. Hutchinson

**Affiliations:** 1 Discipline of Physiology, Adelaide Medical School, University of Adelaide, Adelaide, South Australia 5005 Australia; 2 Australian Research Council Centre of Excellence for Nanoscale BioPhotonics, University of Adelaide, Adelaide, South Australia 5005 Australia

## Abstract

Toll-like receptors (TLRs) are expressed in enteric neurons, glia, gastrointestinal (GI) smooth muscle and mucosa, yet their functional roles in the GI tract are not fully understood. TLRs have been linked to many of the undesirable central effects of chronic opioid administration including hyperalgesia and dependence via activation of central microglia. Opioid-induced bowel dysfunction (OIBD) remains a primary reason for the reduction or withdrawal of opioid analgesics. Morphine-induced inhibition of colonic motility was assessed *in vivo* by GI transit studies and *in vitro* using isolated colons from wildtype (WT) and TLR deficient mice. Morphine slowed movement of ingested content in WT but this retardation effect was attenuated in *TLR4*^*−/−*^ and *TLR2/4*^*−/−*^. In isolated colons, morphine reduced amplitude and frequency colonic migrating motor contractions in both WT and *TLR2/4*^*−/−*^. Electrical field stimulation elicited distal colon relaxation that was potentiated by morphine in WT but not in *TLR2/4*^*−/−*^. Inhibitory junction potentials were of similar amplitude and kinetics in WT and *TLR2/4*^*−/−*^ distal colon and not altered by morphine. Enteric nerve density and proportion of nitrergic nerves were similar in WT and *TLR2/4*^*−/−*^ distal colon. These data suggest an involvement of TLRs in opioid pharmacodynamics and thus a potential interventional target for OIBD.

## Introduction

Opioids such as morphine remain the most effective and widely used analgesics to alleviate moderate to severe pain today. However, it is well recognized that, in addition to exerting potent analgesic actions, the chronic use of opioids can lead to undesirable effects that include tolerance, dependence, atypical pain and bowel dysfunction, with the pain and discomfort from constipation being one of the most common reasons for the reduction or withdrawal of opioid medications in the clinical setting^1^. Despite the length of time opioids have been used, controversy and uncertainty remain regarding the mechanisms by which opioids give rise to these undesirable effects.

In addition to reducing epithelial secretion of fluid into the intestinal lumen, increasing sphincter resting tone, and decreasing rectal sensitivity; opioids cause a reduction in propulsive colonic peristaltic contractions, an increase in non-propulsive tonic contractions and a net increase in colonic muscle tone^2–5^. The actions of morphine on gastrointestinal motility have been attributed to the activation of the classical opioid receptors (μ, κ and δ), with the mu (µ) receptor being of major importance in mediating the anti-transit effects in the large bowel^5,6^. The role of the classical opioid receptors in mediating decreased intestinal and colonic transit effects in response to opioid medications has been based largely on the observation that naloxone, a competitive non-selective antagonist of µ-opioid receptors, suppresses the opioid-driven anti-transit effects^7^. However, studies investigating the effects of opioids on the central nervous system have demonstrated that opioid receptor agonists and antagonists not only interact with the classical opioid (μ, κ and δ) receptors, but also bind to and modulate activity of the innate immune receptors of the Toll-like receptor family, including Toll-like receptor^8–11^.

Interestingly, Toll-like receptors are widely expressed within the gastrointestinal tract, within enteric nerves^12–14^, smooth muscle cells^12,14,15^ and glia^16^ and a role for Toll-like receptors in gut dysmotility has been implicated in a number of gastrointestinal pathologies including irritable bowel syndrome^17^ and inflammatory bowel disease^18,19^. Recently it has been demonstrated that the morphine-induced suppression of colonic transit can be partially mitigated *in vivo* by pre-treatment with a TLR4 antagonist (TAK-242)^20^. This has exciting implications for the potential use of selective TLR antagonists as adjuncts to opioid analgesics and requires further exploration to understand the extent of TLR contribution to the opioid-induced inhibition of gastrointestinal transit and the mechanisms involved. In the current study we have utilised genetic null mutant mice lacking TLR4 or TLR2 and TLR4 receptor subunits to determine, firstly: whether loss of TLR signalling leads to changes in gastrointestinal transit, peristaltic contractions (recorded *in vitro* from intact colon preparations) and post-junctional electrical and mechanical responses to nerve stimulation; and, secondly: whether the effects of morphine on gastrointestinal transit and functional responses recorded from isolated colon are altered as a consequence of TLR deficiency. As it has been hypothesised that lack of TLR signalling during embryonic development can lead to changes in the distribution and density of nitrergic nerves supplying the colon^21,22^, we have expanded our investigation to determine whether any differences in inhibitory responses in the presence of morphine seen in *TLR2/4* deficient mice are due to intrinsic differences with nitrergic transmission and/or attenuated post-junctional responsiveness to nitric oxide. Using immunohistochemistry, we have investigated the expression patterns of neuronal nitric oxide synthase within the colon of adult *TLR2/4*^*−/−*^ mice and have performed functional experiments to determine whether mechanical responses of the colon to the exogenous application of nitric oxide and/or the inhibition of nitric oxide synthase activity are altered in TLR deficient animals.

## Methods

### Animals

BALB/c wildtype mice (*WT*) were obtained from the Animal Resource Centre (Perth, WA, Australia). Mice with null mutations in TLR4 gene (*TLR4*^−/−^), TLR2 and TLR4 genes (*TLR2/4*^*−/−*^) and MyD88 gene (*MyD88*^*−/−*^) which had been back crossed onto a BALB/c background for more than 10 generations were sourced from Prof Akira (Osaka University, Osaka, Japan) and obtained from Prof Paul Foster (University of Newcastle, NSW, Australia). All mice were housed within the facilities provided by Laboratory Animal Services, University of Adelaide, in temperature (23 ± 3 °C) and light/dark cycle (12/12 h) controlled, pathogen-free rooms with standard rodent food and water available *ad libitum*. Mice aged 12–14 weeks were used in *in vivo* gastrointestinal transit studies. For *in vitro* tissue bath and histological experiments colonic tissue was isolated from WT and *TLR2/4*^*−/−*^ animals also aged 12–14 weeks. For *in vitro* experiments, mice were anaesthetized by isoflurane inhalation and culled by cervical dislocation. All experimental procedures were performed in accordance with the University of Adelaide Animal Ethics Guidelines, and approved by the University of Adelaide Animal Ethics Committee.

### Solutions and Drugs

For *in vivo* gastrointestinal transit studies only, a solution of morphine was made in sterile saline (0.9% sodium chloride) and injected subcutaneously (0.1 ml/kg) to achieve final dose of 10 mg/kg. Saline (0.9% sodium chloride) was used as a vehicle control.

The composition of Krebs solution used in *in vitro* mechanical and intracellular microelectrode experiments was (in mM): NaCl 118.0; KCl 4.75; NaHCO_3_ 25.0; glucose 11.0; MgSO_4_ 1.2; NaH_2_PO_4_ 1.0; CaCl_2_ 2.5. Krebs pH was maintained at 7.3–7.4 when bubbled with 95% O_2_–5% CO_2_ at 36.0 ± 0.5 °C.

For *in vitro* mechanical and electrical experiments morphine; atropine, tetrodotoxin (TTX); thromboxane A2, sodium nitroprusside (SNP), nitro-L-arginine LNNA) and nifedipine were each prepared as a stock solution (either in water or ethanol depending on solubility) then diluted to final concentrations in Krebs solution as shown in the text. All drugs were obtained from Sigma (Sydney, NSW, Australia) except morphine, which was obtained from Fauldings Australia (Adelaide, SA, Australia).

### *In vivo* gastrointestinal transit studies

A total of 10 wildtype (WT), 10 *TLR4*^−/−^, 10 *TLR2/4*^−/−^ and 10 *MyD88*^*−/−*^ mice were used in *in vivo* gastrointestinal transit studies. Three days prior to transit experiments mice were individually housed and access to food was removed at 1800 h on the day preceding the experiment. At 0800 the following morning, mice were transferred to individual plastic cages without bedding (allowing visualisation of faecal material) and left to acclimatize to the cage for 1 h. Mice were either administered a subcutaneous (s.c.) injection of 10 mg/kg morphine (5 animals from each group) or saline (5 animals from each group) 30 min before being gavaged (at approximately 0930 h; using a 3 cm, 20 gauge gavaging needle) with 200 μL of an Evans blue (5% Evans blue, 5% gum arabic) marker. Mice were returned to their individual cages and the time taken until Evans blue was seen in faecal droppings was recorded.

### Whole colon organ bath experiments

Following cull, the entire colon was removed and placed in a petri dish filled with warm (30–35 °C) Krebs solution bubbled with a gaseous mixture of 95% O_2_ and 5% CO_2_. A syringe filled with warm (30–35 °C) Krebs solution was inserted into the proximal end of the colon and used to gently eject luminal contents. Mesenteric attachments to the colon were preserved to assist with anchoring the colon to the Sylgard**®**-based organ bath. Within the organ bath, colon preparations were constantly superfused with oxygenated Krebs solution maintained at 36.0 ± 0.5 °C. Contractile forces generated at proximal, mid and distal recording sites along the colon were recorded using 3 independently mounted high-sensitivity isometric recording transducers (AD Instruments MLT0202; Bella Vista, NSW, Australia). Small stainless-steel rakes were inserted at each site and connected to transducers via silk suture thread to enable changes in circumferential tension to be recorded. Passive tension of 10 ± 2 mN was applied to each recording region and preparations left to equilibrate for approximately 1 hour. Analogue signals recorded via transducers were amplified by a quad bridge amp (AD Instruments ML224; Bella Vista, NSW, Australia), before being converted to digital signals using a Powerlab 4/35 data acquisition device (AD Instruments; Bella Vista, NSW, Australia) and recorded for later analysis on a PC running LabChart 7 software (AD Instruments; Bella Vista, NSW, Australia).

### Mechanical studies on isolated distal colon segments

Segments of distal colon (6–8 mm in length) isolated from WT and *TLR2/4*^*−/−*^ mice were mounted in individual chambers of a multi-chamber organ bath (Panlab, AD Instruments, Bella Vista, NSW, Australia). Individual segments (two isolated from each animal) were attached at one end to a fixed mount, orientated in the direction of the longitudinal muscle and secured to an isometric tension transducer using silk suture thread. Electrical field stimulation (EFS) comprising of 30 s duration trains of square wave pulses (0.5 ms pulse duration) at frequencies of 5, 10 and 20 Hz were delivered via stimulation rings located immediately above and below the distal colon segments. Electrical pulses were generated by a Grass S48 stimulator, Grass Instruments; MA, USA). In experiments designed to examine the relaxation responses of isolated distal colon segments (in response to either EFS or exogenous sodium nitroprusside), tissues were pre-contracted with thromboxane A2 (3 × 10^−7^ M), and atropine (10^−6^ M) was added to the circulating Krebs solution to minimise any cholinergic contraction responses.

### Electrophysiological recording of inhibitory junction potentials

Distal colon segments isolated from WT and *TLR2/4*^*−/−*^ mice were opened into flat sheet preparations by cutting parallel to the direction of the longitudinal muscle, close to the mesenteric border. Mucosal layers were then carefully removed by sharp dissection and the remaining *tunica muscularis* pinned, circular muscle layer uppermost, to the Sylgard-lined base of a small (40 mm diameter) recording chamber. Colon segments were superfused with Krebs solution constantly bubbled with a mixture of 95% O_2_ and 5% CO_2_ and maintained at 36.0 °C ± 0.5 °C.

Membrane potentials were recorded via high-resistance (80–120 MΩ) electrodes filled with 3 M KCl. Analogue voltage signals were pre-amplified by an Axoclamp-2B amplifier, digitized and relayed to a PC computer running Axoclamp 9.0 software (Axon Instruments; now Molecular Devices). Pulses of EFS (0.5 ms duration square wave pulses; 1 to 20 Hz in 1 s trains) were generated by a Grass S48 stimulator and delivered to distal colon segments via platinum stimulating wires positioned across preparations.

### Immunohistochemistry

Distal colon segments isolated from WT and *TLR2/4*^*−/−*^ mice were opened along the mesenteric border and pinned securely to a Sylgard-coated dissecting dish containing PBS (0.01 M). Mucosal layers were removed via sharp dissection, and the remaining muscle layers fixed in paraformaldehyde (4% in 0.01 M PBS; pH 7.2; 10 min). After rinsing (3 × 20 min washes) in PBS, tissues were incubated in bovine serum albumin (BSA; 1% in 0.01 M PBS at room temperature (RT); 1 hour) to reduce non-specific antibody binding. Tissues were sequentially incubated with antibodies to human neuronal protein HuC/HuD (Hu; Molecular Probes; Clone 16A1; mouse IgG2b, biotin conjugate) and neuronal nitric oxide synthase (gift from Piers Emson, Cambridge; Clone K205; sheep IgG), each diluted in a weak solution of Triton X-100 (0.03% in 0.01 M PBS; 48 hours). Immunoreactivity for Hu and nNOS were detected using Alexa Fluor 488 Streptavidin and Alexa Fluor 568**®** donkey anti-sheep IgG (Invitrogen) respectively, each incubated sequentially at RT in 0.01 M PBS; 1 hour) and rinsed (3 × 20 min washes) between incubations. Negative control tissues were prepared by omitting either primary or secondary antibodies from the incubation solution. Tissues were examined and images captured using a Leica SP5 laser scanning confocal microscope with appropriate excitation wavelengths. For each whole-mount distal colon preparation, obtained from an individual animal, 3 non-adjacent, randomly selected images (at 20× magnification) were captured by performing z-series scans at 1 µm intervals through the circular muscle layer and myenteric plexus region. Stacked digital images typically consisted of 20–25 × 1 µm z-series scans.

Following image acquisition, an investigator, who was blinded to animal group allocation of the images, reprojected Z-series scans of the myenteric plexus region and counted the number of Hu and nNOS positive neuron cell bodies in each image (image area at 40× magnification = 390 × 390 µm = 0.152 mm^2^). The number of Hu immunopositive cell bodies within the 3 images were summed to obtain a value for the ‘number of Hu positive neurons’ whilst the number of cell bodies immunopositive for both NOS and HuC/D immunopositive was used to calculate the proportion of NOS positive neurons. Average ganglion area and the number of Hu positive and nNOS positive cells per ganglion were also determined for wildtype control and *TLR2/4*^*−/−*^ animals. For this, 20 ganglia from each animal group were randomly selected by a blinded investigator. The average area occupied by individual ganglia was determined using ImageJ software (NIH) by creating an outline around the ganglionic structure.

To determine the density of nNOS positive enteric terminals, composite confocal micrographs were constructed by projecting z-series scans collected through the circular muscle layer of each whole mount preparation. The number of terminals crossing 3 evenly spaced 100 µm transect lines were counted and averaged to give an averaged terminal density value for each animal. Data from 5 animals in each group (WT and *TLR2/4*^*−/−*^) were averaged and expressed as mean ± SEM.

### Statistics

For *in vivo* transit studies, one-way ANOVA followed by a Tukey’s multiple comparisons test was used to determine statistical differences in entire gut transit time between groups. For *in vitro* motility studies, CMMC parameters and the characteristics of mechanical and electrical responses to electrical field stimuli were compared between animal groups (and/or between drug applications) using student’s t-tests or ANOVA (with Tukey’s multiple comparison) where appropriate.

### Data availability

The datasets generated during and/or analysed during the current study are available from the corresponding author on reasonable request.

## Results

### Systemic morphine fails to slow gastrointestinal transit time in TLR2/4, TLR4 and MyD88 deficient mice compared to wildtype (WT) mice

**Figure 1:**
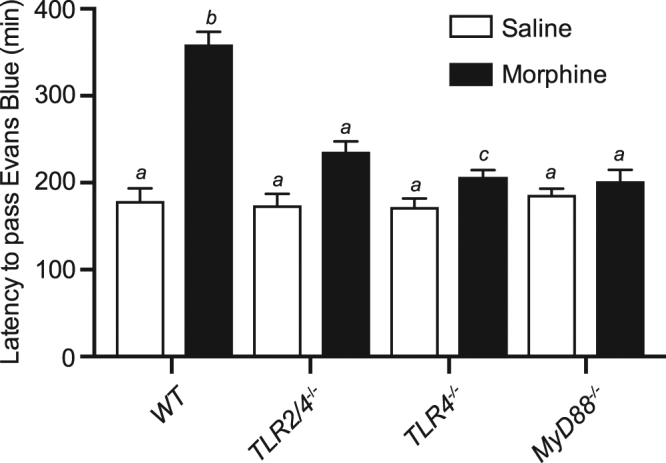
Bar graph summarising latency (in minutes from time of gavage) for Evans Blue dye to be visible in the faecal droppings of WT, *TLR2/4*^*−/−*^, *TLR4*^*−/−*^ and *MyD88*^*−/−*^ mice with pre-injection of saline (white) or morphine (black). Data is expressed as mean ± SEM. Bars not sharing the same letter annotations are statistically different (*P* < 0.05; *n* = 5 per group).

The latency for the Evans blue dye to appear in fecal material from WT mice receiving subcutaneous injection of saline (vehicle control) was 179 ± 14 min (n = 6). Under the same control conditions there was no significant difference in the latency to pass Evans Blue dye in *TLR2/4*^*−/−*^ (173 ± 10 min), *TLR4*^*−/−*^ (175 ± 13 min) or *MyD88*^*−/−*^ (187 ± 7 min) mice compared to WT controls (Fig. 1). Following administration of morphine, the latency for Evans blue dye to appear in the stool was increased to 360 ± 14 min for WT (p < 0.0001 compared to saline) and to 208 ± 7 min for *TLR2/4*^*−/−*^ mice (p < 0.01 compared to saline). The average latency for the appearance of Evans blue dye in the stool following administration of morphine was 236 ± 11 min for *TLR4*^*−/−*^ and 202 ± 13 min for *MyD88*^*−/−*^ groups, which were not significantly different to latency values following saline only within the same animal groups and significantly shorter than for WT in the presence of morphine (Fig. 1).

### Colonic Migrating Motor Contractions (CMMCs) within isolated *TLR2/4*^*−/−*^ and WT colons are similar in amplitude and frequency

**Figure 2:**
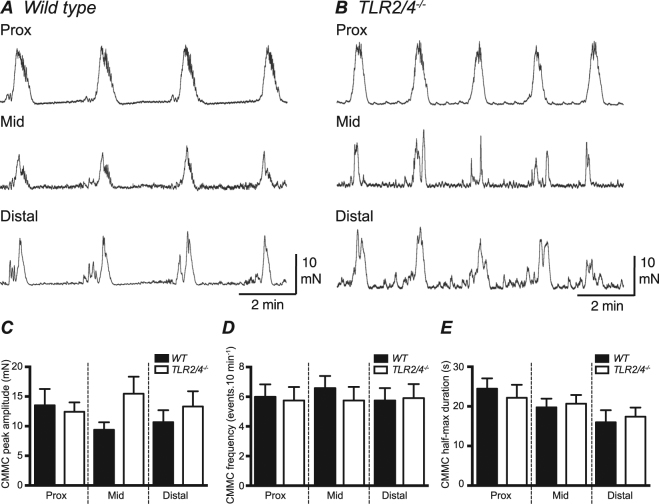
Comparison of colonic migrating motor complexes recorded *in vitro* from isolated WT and *TLR2/4*^*−/−*^ colon. Typical digital recordings of contractions occurring at proximal, mid and distal sites of WT (**A**) and *TLR2/4*^*−/−*^ (**B**) colon. Mean CMMC contraction parameters of amplitude (**C**), frequency (**D**) and half-max duration (**E**) values for each region of the colon (proximal, mid and distal) from WT (black bars) and *TLR2/4*^*−/−*^ (white bars).

A series of *in vitro* mechanical experiments were performed to determine if loss of both TLR2 and TLR4 receptor subunits altered the kinetics and characteristics of migrating contractions recorded from isolated colons. When tension changes were simultaneously recorded from proximal, mid and distal regions, large amplitude, propagating contractions (previously termed colonic migrating motor contractions^23^; CMMCs were recorded from WT (Fig. 2A) and *TLR2/4*^*−/−*^ (Fig. 2B) colon. Mean CMMC contraction parameters (amplitude, frequency and half-max duration values) for each region of the colon (proximal, mid and distal) were similar (P > 0.05) between WT and *TLR2/4*^*−/−*^ groups (summarized in Fig. 2C–E).

### Morphine inhibits CMMC frequency and amplitude in whole WT and *TLR2/4*^*−/−*^ colon

**Figure 3:**
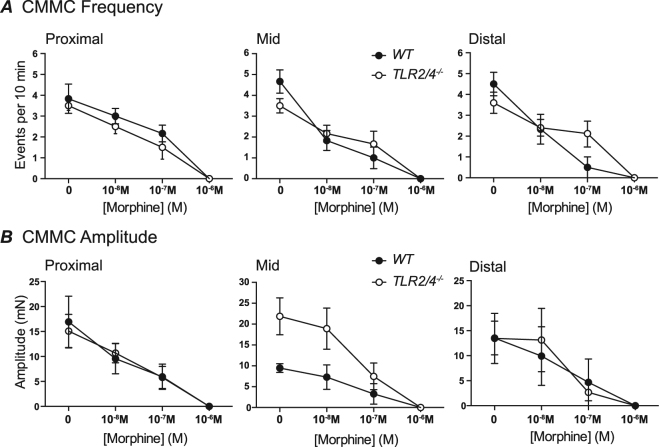
Graphs summarising the mean frequency (**A**) and amplitude (**B**) of colonic migrating motor complexes at proximal, mid and distal sites of WT (black circle) and *TLR2/4*^*−/−*^ (open circle) colon following increased morphine concentrations.

The effect of increasing morphine concentration on CMMC activity recorded from WT and *TLR2/4*^*−/−*^ colon *in vitro* was examined. CMMC frequency and amplitude were reduced at proximal, mid and distal sites of both WT and *TLR2/4*^*−/−*^ colon as the concentration of morphine was increased (Fig. 3). At the distal end of WT colons the frequency of CMMCs was significantly reduced by 10^−7^ M morphine (i.e. from 4.5 ± 0.5 events per 10 min to 0.5 ± 0.5 events per 10 min (p < 0.001; n = 6). In contrast, the same concentration of morphine did not produce a statistically significant reduction of CMMC frequency in *TLR2/4*^*−/−*^ distal colon (i.e. 3.7 ± 0.4 events per 10 min prior to administration of morphine and 2.0 ± 0.7 events per 10 min in presence of 10^−7^ M morphine (p = 0.24; n = 6). It was noted that for 5 of 6 WT animals (83%), 10^−7^ µM morphine was sufficient to abolish CMMC activity at the distal end, whereas the same concentration of morphine only abolished CMMC activity at the distal end of 2 of 6 *TLR2/4*^*−/−*^ colon preparations (33%).

### Small amplitude contractions are potentiated by TTX in WT but not *TLR2/4*^*−/−*^ distal colon

**Figure 4:**
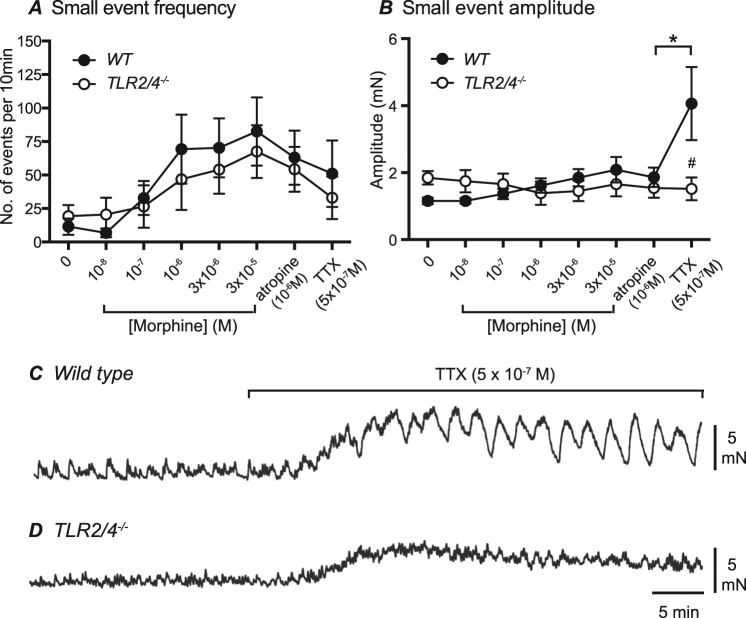
Summarised frequencies (**A**) and amplitudes (**B**) of small contractile events in WT (black circle) and *TLR2/4*^*−/−*^ (open circle) colon in the presence of increased concentrations of morphine, atropine and TTX. Typical digital recordings of small amplitude contractions occurring at the distal sites of WT (**C**) and *TLR2/4*^*−/−*^ (**D**) colon following TTX application. *Denotes p < 0.05 between small event amplitudes pre- and post-TTX in WT animals. #Denotes p < 0.05 between WT and *TLR2/4*^*-/-*^ small event amplitudes in presence of TTX.

At distal recording sites of WT colon, under control conditions, small amplitude (1.2 ± 0.1 mN), high frequency (11.5 ± 2.3 events per 10 min) contractions were commonly interposed between larger migrating contractions. In *TLR2/4*^*−/−*^ distal colon these smaller amplitude contractions were of similar amplitude (average amplitude 1.8 ± 0.2 mN) and frequency (19.3 ± 4.2 events per 10 min recording period) to WT controls. In response to an increase in morphine concentration (from 10^−8^ M to 3 × 10^−5^ M), the frequency of small contractile events increased in both WT and *TLR2/4*^*−/−*^ colon (Fig. 4A) without significant changes in amplitude in either animal group (Fig. 4B). To determine if blockade of muscarinic receptors would alter or abolish small amplitude contractions, the muscarinic receptor antagonist atropine (10^−6^ M) was applied in the continued presence of morphine. For both animal groups, small amplitude contractions persisted in the presence of atropine with no change of amplitude or frequency. Application of the fast sodium channel blocker, tetrodotoxin (TTX; 5 × 10^−7^ µM), to inhibit neural action potentials (and thus presumably reduce tonic neurotransmitter release) was associated with an increase in resting tone (~2 mN) and an increase in the amplitude of small contractile events in WT distal colon (i.e. from an average amplitude of 1.9 ± 0.3 mN in atropine to 4.1 ± 1.0 mN following application of TTX; n = 5; p = 0.05; Fig. 4B,C). TTX also produced a small increase in resting tone in *TLR2/4*^*−/−*^ colon but was not associated with an increase in the amplitude of the small contractile events (1.5 ± 0.3 mN in atropine and 1.5 ± 0.3 mN in TTX (p = 0.88; n = 6; Fig. 4B and D).

### Relaxations elicited by electrical field stimulation are potentiated by morphine in WT but not in *TLR2/4*^*−/−*^ distal colon

**Figure 5:**
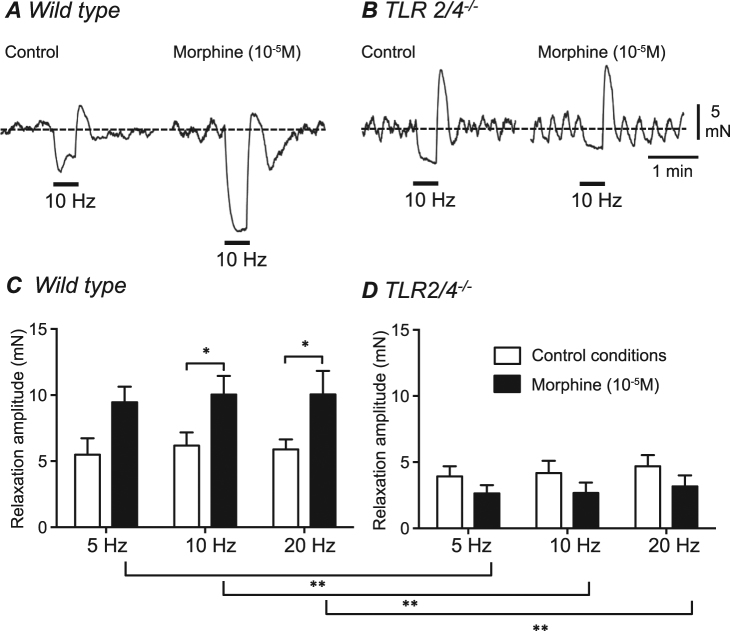
Comparison of EFS induced relaxation responses recorded *in vitro* from isolated segments of distal colon from WT (**A**) and *TLR2/4*^*−/−*^ (**B**). A summary of the mean relaxation amplitude in distal colon isolated segments following EFS stim (5, 10 & 20 Hz) in the absence (white bars) or presence of morphine (10^−5^ M) (black bars) from WT (**C**) and *TLR2/4*^*−/−*^ (**D**). *denotes p < 0.05; **denotes p < 0.01.

A series of *in vitro* experiments were performed on isolated segments of WT and *TLR2/4*^*−/−*^ distal colon to investigate the effect of morphine on colonic tone and contractility patterns in the absence and presence of TLR receptor subunits. In order to resolve relaxation responses of distal colon segments more clearly, following tissue equilibration, thromboxane A2 (3 × 10^−7^ M) and atropine (1 × 10^−6^ M) were added to the tissue perfusate and maintained throughout the duration of the experiment. The increase in tone elicited by thromboxane A2 did not differ between WT (n = 6) and *TLR2/4*^*−/−*^ (n = 8) distal colon preparations (p = 0.84). In the presence of thromboxane A2 (control conditions), electrical field stimulation using 30 sec trains of pulses (pulse duration 0.5 ms; delivered at 10 Hz) elicited relaxation of WT distal colon segments (Fig. 5A). Addition of morphine (10^−5^ M) produced a small increase in basal tone (average increase of 1.3 ± 0.2 mN; not shown) and a potentiation of EFS-evoked relaxation (Fig. 5A). In *TLR2/4*^*−/−*^ distal colon segments, 10 Hz EFS also elicited small relaxations in control conditions (Fig. 5B). Addition of morphine (10^−5^ M) to *TLR2/4*^*−/−*^ distal colon segments also typically produced an increase in basal tone (average increase of 2.4 ± 0.8 mN); however, unlike WT controls, there was no potentiation of *TLR2/4*^*−/−*^ distal colon relaxations in response to 10 Hz EFS in the presence of morphine (Fig. 5B). Amplitudes of relaxation responses elicited from isolated, pre-contracted distal colon segments in response to a range of stimulation frequencies are summarized in Fig. 5C,D. In WT segments, morphine significantly potentiated the amplitude of relaxations evoked by 10 and 20 Hz EFS (Fig. 5C; p < 0.05 compared to prior to addition of morphine). This potentiation of relaxation amplitude by morphine was not seen in distal colon segments isolated from *TLR2/4*^*−/−*^ animals at any stimulation frequency tested (Fig. 5D).

**Figure 6:**
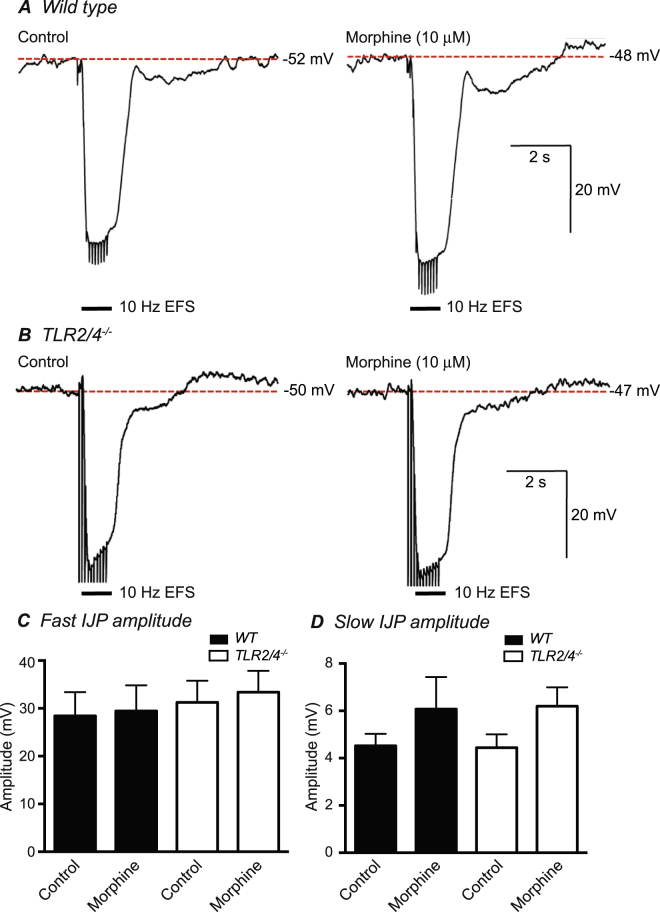
Comparison of inhibitory junction potentials (IJPs) evoked by 10 Hz EFS recorded *in vitro* in the presence or absence of morphine (10^−5^ M) from *WT* (**A**) and *TLR2/4*^*−/−*^ (**B**) distal colon preparations. A graph summary showing the mean amplitude of fast (**C**) and slow (**D**) component of IJPs from *WT* (black bars) and *TLR2/4*^*−/−*^ (white bars) in the absence or presence of morphine (10^−5^ M).

### Resting membrane potentials and post-junctional electrical responses elicited by EFS are similar in WT and *TLR2/4*^*−/−*^ colon

Resting membrane potentials of circular smooth muscle cells within distal segments of WT and *TLR2/4*^*−/−*^ colon were not different, averaging −54.3 ± 1.2 mV and −53.0 ± 1.0 mV respectively (n = 5 for each animal group; p = 0.78). Addition of morphine (10^−5^ M) elicited a small (~3 mV) depolarization of membrane potential in both animal groups. Inhibitory junction potentials (IJPs) evoked by 10 Hz EFS were of similar amplitude and kinetics in WT and *TLR2/4*^*−/−*^ distal colon preparations (Fig. 6A,B). In WT colon the initial, rapid onset component of the IJP averaged 28.4 ± 4.9 mV in amplitude and was unaffected by morphine (29.5 ± 5.4 mV post-morphine; n = 5; p = 0.54). The slower, later onset, IJP component averaged 4.5 ± 0.5 mV prior to and 6.1 ± 1.3 mV following the addition of morphine (p = 0.31). Fast IJPs elicited in *TLR2/4*^*−/−*^ distal colon by 10 Hz EFS averaged 31.3 ± 4.5 mV prior to, and 33.4 ± 4.4 mV following morphine (p = 0.43; n = 5), whilst the slow IJP averaged 4.4 ± 0.6 mV and 6.2 ± 0.8 mV before and after morphine respectively (p = 0.11; n = 5; Fig. 6C,D).

### Immunohistochemical analysis reveals similar numbers of enteric nerve cell bodies and proportion of nNOS-immunopositive neurons in WT and *TLR2/4*^*−/−*^ distal colon

**Figure 7:**
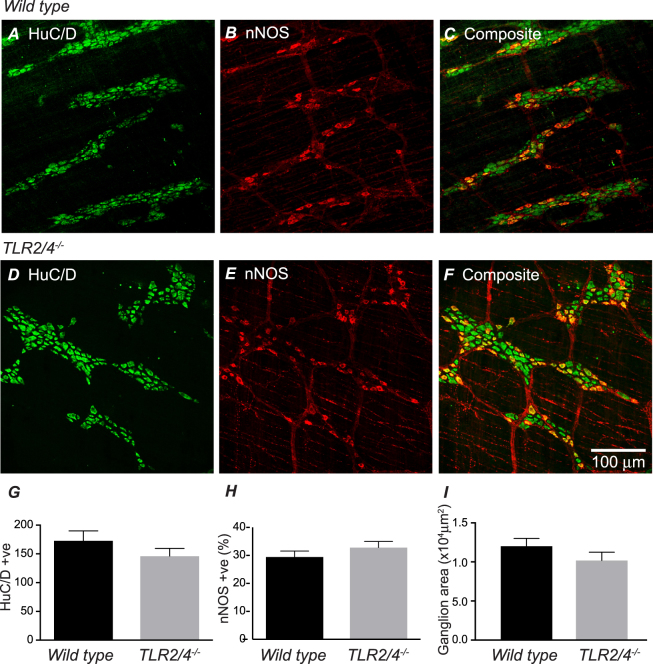
Immunohistochemical localisation of HuC/D (**A**,**D**) and neuronal NOS (**B**,**E**) in the myenteric plexus and circular muscle layer of distal colon from *WT* (**A**–**C**) and *TLR2/4*^*−/−*^ (**D**–**F**). The composite images (**C**,**F**) illustrate the proportion of HuC/D positive neurons that also express nNOS. Scale bar in F applies to all panels. Summary graphs compare number of HuC/D positive neurons (**G**), proportion of nNOS positive neurons (**H**) and ganglion area (**I**) in distal colon of *WT* and *TLR2/4*^*−/−*^.

To determine whether attenuated inhibitory responses of *TLR2/4*^*−/−*^ distal colon observed in the presence of morphine were associated with decreased populations of enteric neurons, distal colon segments were labelled using antibodies to the pan-neuronal protein HuC/D and neuronal nitric oxide synthase (nNOS) (Fig. 7). Analysis of confocal micrographs revealed the number of HuC/D immunopositive cell bodies did not differ significantly between WT and *TLR2/4*^*−/−*^ distal colon segments (WT: 173 ± 17 and *TLR2/4*^*−/−*^: 146 ± 13 cell bodies within 3 randomly selected 40× images from 5 animals in each group; p = 0.21; Fig. 7A–G). The proportion of NOS immunopositive nerve cell bodies within wildtype distal colon images was 29.5 ± 2.0% whilst this was 32.8 ± 2.1% in *TLR2/4*^*−/−*^ (n = 5 animals, 3 images per animal; p = 0.26; Fig. 7H).

The mean number of ganglia per wild type image (covering area of 0.15 mm^2^) was 2.05 ± 0.2 (min 1; median 2; max 4). In comparison, the average number of ganglia observed in images acquired from TLR2/4 KO distal colon the density of ganglia was 1.7 ± 0.3 (min 1; median 1.5; max 3; p = 0.3 for mean comparisons). Average ganglion area in wildtype preparations was 10,188 ± 1056 um^2^, and did not differ significantly in *TLR2/4*^*−/−*^ (12,031 ± 972 um^2^; P = 0.21; n = 20 ganglia per animal group; Fig. 7I). The average number of Hu positive cell bodies located within ganglia of wildtype and *TLR2/4*^*−/−*^ distal colon was almost identical, with 30.8 ± 3.2 cell bodies per ganglion in wild type and 30.9 ± 2.9 cell bodies per ganglion in *TLR2/4*^*−/−*^ animals (P = 0.97). Wildtype ganglia contained on average 13.2 ± 1.5 nNOS positive cell bodies, whilst 14.1 ± 1.9 cell bodies per ganglion were nNOS positive in *TLR2/4*^*−/−*^ distal colon (P = 0.7).

In addition, the density of nNOS immunopositive nerve terminals (WT: 5.6 ± 1.3 and *TLR2/4*^*−/−*^: 4.9 ± 0.7 per 100 μm transect line; n = 5 per group; 3 transect lines per n) did not differ significantly between animal groups (p = 0.26; data not shown).

### Relaxation responses to high concentrations of the nitric oxide donor, sodium nitroprusside, are reduced in *TLR2/4*^*−/−*^ compared to WT distal colon

**Figure 8:**
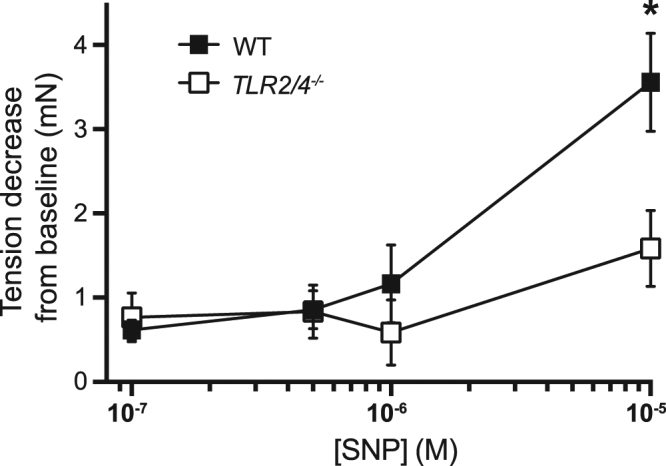
Summarised tension decreases (muscle relaxations) elicited by graded increases in the concentration of the nitric oxide donor, sodium nitroprusside (SNP) in distal colon segments isolated from wild type (*WT*) and *TLR2/4*^*−/−*^ mice. *Denotes p < 0.05 for comparison between WT and *TLR2/4*^*−/−*^.

Attenuated EFS-evoked relaxation responses in *TLR2/4*^*−/−*^ distal colon in the presence of morphine could potentially be explained by a decreased responsiveness of post-junctional effector cells (i.e. smooth muscle cells, intramuscular interstitial cells of Cajal and/or PDGFRα positive cells) to the major inhibitory transmitter nitric oxide. Hence, a series of experiments was conducted to determine whether *TLR2/4*^*−/−*^ distal colon preparations relaxed similarly to WT distal colon in response to exogenous application of the nitric oxide donor sodium nitroprusside (SNP). Relaxations to cumulative increases in SNP concentration (from 10^−7^, 5 × 10^−7^, 10^−6^ through to 10^−5^ M) in WT and *TLR2/4*^*−/−*^ distal colon segments were compared. Relaxations of distal colon segments in response to increasing concentrations of sodium nitroprusside are summarised in Fig. 8. Relaxations elicited by lower concentrations of SNP (10^−7^ to 10^−6^ M) were not significantly different between WT and *TLR2/4*^*−/−*^ groups. However, when the concentration was increased to 10^−5^ M, relaxations of WT distal colon were significantly larger in amplitude than *TLR2/4*^*−/−*^ distal colon (Fig. 8) suggesting a decreased sensitivity of *TLR2/4*^*−/−*^ distal colon to high concentrations of nitric oxide.

### Nitric oxide synthesis inhibition increases CMMC activity in WT and *TLR2/4*^*−/−*^ distal colon

Pharmacological blockade of nitric oxide production (using the nitric oxide synthase inhibitor nitro-L-arginine L-NNA) produces an acute increase in the amplitude and frequency of murine colonic migrating motor contractions^23^. Therefore, it was hypothesized that if nitrergic innervation or the post-junctional responsiveness to nitric oxide at the distal end of *TLR2/4*^*−/−*^ colon were compromised then application of the nitric oxide synthase inhibitor nitro-L-arginine (L-NNA) would have reduced effect in these preparations. A series of *in vitro* mechanical experiments were performed to address this hypothesis by comparing the effect of pharmacological nitric oxide synthase inhibition on CMMC activity recorded from intact WT and *TLR2/4*^*−/−*^ colons. In WT colon, L-NNA (100 μM) produced an acute increase in CMMC frequency (averaged across proximal, mid and distal sites) from 7.3 ± 0.7 to 10.8 ± 0.6 events per 10 min (i.e. frequency post-LNNA was 155 ± 16% compared to pre-LNNA frequency; n = 6; p < 0.01). L-NNA elicited a similar increase in CMMC frequency in *TLR2/4*^*−/−*^ colon from an average of 7.9 ± 1.2 events to 11.4 ± 1.1 events per 10 min; (i.e. frequency post-LNNA was 153 ± 18% compared to pre-LNNA frequency; n = 6; p < 0.01). For both animal types (WT and *TLR2/4*^*−/−*^), the effect of L-NNA on CMMC amplitude and duration were more pronounced within the distal colon than at proximal and mid sites. In WT distal colon L-NNA (10^−4^ M) caused CMMC amplitude and duration to increase to 168 ± 36% and 160 ± 27% of control values respectively (n = 6). Similarly, in *TLR2/4*^*−/−*^ distal colon, CMMC amplitude and duration were increased to 211 ± 22% and 172 ± 33% of control values respectively (n = 6). No significant differences in response to L-NNA were evident between animal strains. Example traces showing the effect of L-NNA on CMMC activity recorded from wildtype and *TLR2/4*^*−/−*^ colon are provided in Supplementary Figure 1. Graphs summarising the acute (after 5 mins) and prolonged (after 30 mins) effects of L-NNA on amplitude, frequency and duration of CMMCs and basal tension recorded from proximal, mid and distal sites of WT and TLR2/4−/− colon are shown in Supplementary Figure 2.

## Discussion

We have found that the inhibitory action of morphine to retard movement of ingested content along the gastrointestinal tract is substantially reduced in genetic mutant mice lacking TLR2 and TLR4 receptor subunits and when their MyD88 dependent signalling is absent. This observation is of substantial interest given the high prevalence of opioid-induced bowel dysfunction in a clinical setting, and the seemingly well-accepted paradigm that the inhibitory effect of morphine on GI motility is mediated exclusively by the classical μ, δ and κ opioid receptors. In an attempt to explore the cellular and molecular origins of this impact of TLR signalling on GI opioid pharmacodynamics we demonstrated that naive *in vivo* (GI transit time) and migrating motor contractions (CMMCs) of isolated colon did not differ between *TLR2/4*^*−/−*^ and WT mice, and *in vitro* CMMCs in both strains were attenuated by morphine. However, *TLR2/4*^*−/−*^ mice did demonstrate significantly different sensitivity to potentiation of small amplitude contractions when tetrodotoxin was applied and EFS-induced relaxations were reduced. These results pointed towards possible differences in nitrergic capacity in the genetic absence of TLR2/4, but resting inhibitory tone and counts of nNOS positive enteric nerve bodies were similar between WT and *TLR2/4*^*−/−*^ mice. Pharmacological manipulation of the nitrergic system also failed to differentiate WT and *TLR2/4*^*−/−*^ mice, except at high concentrations of the nitric oxide donor suggesting that *TLR2/4*^*−/−*^ mice may be more resistant to extensive nitrergic inhibition.

In control *in vivo* transit experiments (in which saline rather than morphine was administered), the time taken for ingested content to move through the gastrointestinal tract of WT compared to that of TLR deficient animals did not differ, confirming that, under normal physiological conditions, TLR2/4 receptors are not fundamentally required for the movement of ingested content through the GI tract. Furthermore, *in vitro* mechanical tension recordings made from proximal, mid and distal sites of isolated WT and *TLR2/4*^*−/−*^ colons revealed that, under control conditions, the frequency, amplitude or duration of colonic migrating motor contractions (CMMCs) did not differ between WT and TLR deficient animals. Thus, despite the differential effect of morphine on transit through the entire GI tract, neither TLR2 nor TLR4 receptor subunits are critical for the development *or* operation of the appropriate neuromuscular machinery required for propulsive colonic motor patterns to occur under basal conditions.

CMMCs are large amplitude, phasic propulsive contractions, widely accepted as being the product of the autonomous activity of the enteric plexus – they are known to occur in the absence of input from extrinsic parasympathetic and sympathetic fibres and thus can be readily recorded from isolated colon preparations^24–27^. In isolated intact colon preparations, morphine reduced the amplitude and frequency of colonic migrating motor contractions (CMMCs) in both WT and *TLR2/4*^*−/−*^ mice in a dose dependent manner – although interestingly higher concentrations of morphine were typically required to abolish CMMC activity at the distal end of TLR deficient colon suggesting that deficiency of TLR2/4 may render the terminal portions of the bowel less sensitive to high concentrations of morphine.

Loss of TLR receptor subunits (and MyD88, a functional adaptor protein in the TLR receptor complex) was found to protect against morphine-induced retardation of transit *in vivo*; yet CMMCs recorded from isolated WT and TLR deficient colons were similarly inhibited by low to moderate concentrations of morphine. This differential effect of morphine in intact animals versus isolated colon segments points towards a possible involvement of TLR signalling pathways extrinsic to the colon in the morphine-induced inhibition of transit *in vivo*. Although it has been demonstrated that intracerebral and microinjections of morphine into the periaqueductal gray can produce inhibition of intestinal transit supporting the notion that a centrally-mediated modulation of GI motility is possible^28^ the more widely accepted paradigm is that morphine-induced retardation of gastrointestinal transit predominantly involves peripheral μ opioid receptors, expressed on intrinsic enteric neurons. Inhibition of gastrointestinal peristalsis is thought to occur primarily as a result of the presynaptic inhibition of excitatory cholinergic neurons within the myenteric plexus^29,30^. In support of the peripherally mediated action of morphine on intestinal transit, methylnaltrexone, a selective antagonist of peripherally expressed opioid receptors, has been shown to be effective in reducing the delay in oral-caecal transit and eliciting laxation responses in cancer patients^31^. Further experiments will be required to elucidate whether the differential effect of morphine that accompanies loss of TLR receptor signalling is a result of peripherally versus centrally located receptors, or a combination of both.

Electrical field stimulation (with parameters set to selectively stimulate nerves) elicited small relaxations of both WT and *TLR2/4*^*−/−*^ distal colon. Application of morphine increased basal tone irrespective of TLR expression, but was only found to significantly potentiate relaxation responses in WT and not *TLR2/4*^*−/−*^ colon. Thus, in the presence of morphine, relaxation responses to field stimulation were significantly larger in WT animals than *TLR2/4*^*−/−*^ colon. It is unclear whether the attenuated relaxation response elicited by nerve stimulation would be sufficient to explain the substantially reduced effect of morphine on total GI transit. However, the differential effect of morphine in TLR intact and deficient colons prompted a number of questions for further investigation, some of which have been addressed by the current study:

It has previously been hypothesised that lack of TLR signalling during embryonic development leads to reduced nitrergic innervation of the colon^21^. We investigated whether reduced relaxation responses of TLR distal colon observed in the presence of morphine were associated with a reduction in the distribution and density of nitrergic motor nerve fibres using nNOS immunohistochemistry. We found no difference in the density or distribution of nNOS immunopositive neuronal cell bodies and nerve terminals within the distal segment of *TLR2/4*^*−/−*^ deficient colon compared to WT controls. Our findings contrast with previous reports of reduced NADPH diaphorase and nNOS immunostaining in the proximal colon of animals deficient in TLR4 (i.e. *TLR4*^*Lps-d*^ and *TLR4*^*−/−*^ mice) or an essential adaptor protein in TLR4 signalling pathway (i.e. *Myd88*^*−/−*^ mice)^21^. It is unclear whether this difference is due to variation in the TLR deficient strains (the mice used in the current study lacked both TLR2 and TLR4 receptors); the anatomical regions (proximal versus distal regions) or the age of the animals investigated (here we used animals 12–14 weeks of age versus 4 weeks in the previous study). Nonetheless, our findings here do not support the hypothesis that TLR signalling is critical for enteric nitrergic nerve survival.

One possible explanation for our findings is that loss of TLR2/4 signalling has led to changes in the responsiveness of post-junctional effector cells (i.e. smooth muscle cells and interstitial cells of Cajal) to inhibitory neurotransmitters such as nitric oxide and that this difference is unmasked in the presence of morphine. In the current study, we sought to determine whether the loss of TLR was associated with (a) a reduced sensitivity to exogenously applied nitric oxide and (b) an attenuated pro-kinetic effect of nitric oxide synthesis blockade. SNP was found to relax both WT and TLR deficient colon tissues to a similar extent when the lower range of concentrations of SNP were applied. However, relaxations to a higher (10 μM) concentration SNP were reduced in *TLR2/4*^*−/−*^ colon suggesting that the responsiveness of the colonic muscle to nitric oxide is compromised with loss of TLR signalling. This concept needs to be further explored to help identify the mechanism responsible for the reduced NO response in colon tissue lacking TLR2 and 4 receptors. Blocking nitric oxide synthesis via application of the nitric oxide synthase (NOS) inhibitor L-NNA (which inhibits all isoforms of NOS; inducible, neuronal and endothelial), produced a similar increase in tone, CMMC frequency, amplitude and duration of CMMCs recorded from the distal end of WT, *TLR4*^*−/−*^ and *TLR2/4*^*−/−*^ animals; suggesting that, when isolated from the animal, the intrinsic motility patterns and the response to pharmacological inhibition of nitrergic input to the colon does not differ significantly regardless of the presence or absence of TLR subunits.

Our findings suggest that TLR receptors may be important functional components of the opioid receptor signalling pathway, with loss of TLR expression altering the extent of GI transit retardation produced by morphine. Interestingly it has also been shown that some of the inhibitory actions of morphine on guinea pig colonic transit (but not small intestine motility) can be mitigated by the acute pharmacological inhibition of TLR receptors using the TLR receptor antagonist TAK-242^20^. The finding that TAK-242 administration reduced the inhibitory actions of morphine on colonic propulsion and transit suggests that an acute blockade of TLR receptors is sufficient to provide some protection against opioid-induced bowel dysmotility. One possibility is that a pharmacodynamic interaction occurs between opioid receptors and toll-like receptor subunits, with loss of the latter in TLR deficient mutant animals causing disruption to cell signalling cascades elicited upon opioid agonist binding. It is unclear whether this type of interaction is unique to TLR and opioid receptors or whether similar interactions exist between TLR subunits and other G-protein coupled receptors.

We have determined that whilst the inhibitory effect of morphine on GI transit is attenuated in TLR deficient animals, we report relatively subtle differences with regards to the responses of isolated WT and TLR deficient colon preparations responses to bath application of morphine. It remains possible that the differential effect of morphine in wild type versus TLR deficient animals in *in vivo* transit studies was due to actions of morphine on GI regions other than the colon (e.g. stomach, small intestine and rectum). In organ bath experiments, contractile activity measurements were taken from sites proximal to the rectum. Despite its short length in mice, the rectum is thought to contribute significantly to the opioid-induced retardation of gastrointestinal transit, previously tested using the bead expulsion method^32^. Thus, it is possible that removal of the very terminal rectum segment in isolated colon experiments contributed to the more modest differences observed between TLR deficient and intact colon compared to the substantial differences in whole GI transit observed in *in vivo* experiments.

## Conclusion

Our findings demonstrate that opioid pharmacodynamic effects in the gastrointestinal tract are impacted by TLR2 and TLR4 expression. Clear differences in the extent of morphine-induced retardation of transit between WT and TLR deficient animals was observed, with *in vivo* transit being substantially less delayed after morphine administration in TLR deficient animals. Differences in response to morphine between isolated *TLR2/4*^*−/−*^ and WT colon preparations were relatively subtle, suggesting that *in vivo* transit differences may involve factors extrinsic to the colon itself. These data reinforce the breadth of opioid pharmacodynamic actions in which TLRs are involved and point to a novel pharmacological target worthy of further investigation to intervene in unwanted opioid GI disturbances.

## Electronic supplementary material

Supplementary figures
