# Supplementary material for: Differential effect of morphine on gastrointestinal transit, colonic contractions and nerve-evoked relaxations in Toll-Like Receptor deficient mice
Source: Sci Rep. 2018 Apr 12;8:5923. doi: 10.1038/s41598-018-23717-4 (PMC5897409; doi:10.1038/s41598-018-23717-4)
Supplement: Supplementary file 1 — Supplementary figures [file 41598_2018_23717_MOESM1_ESM.pdf]

# Differential effect of morphine on gastrointestinal transit, colonic contractions and nerve-evoked relaxations in Toll-Like Receptor deficient mice

*Elizabeth A. H. Beckett[1], Vasiliki Staikopoulos[1, 2] & Mark R Hutchinson[1, 2]

## Supplementary Information

**Supplementary Figure 1**

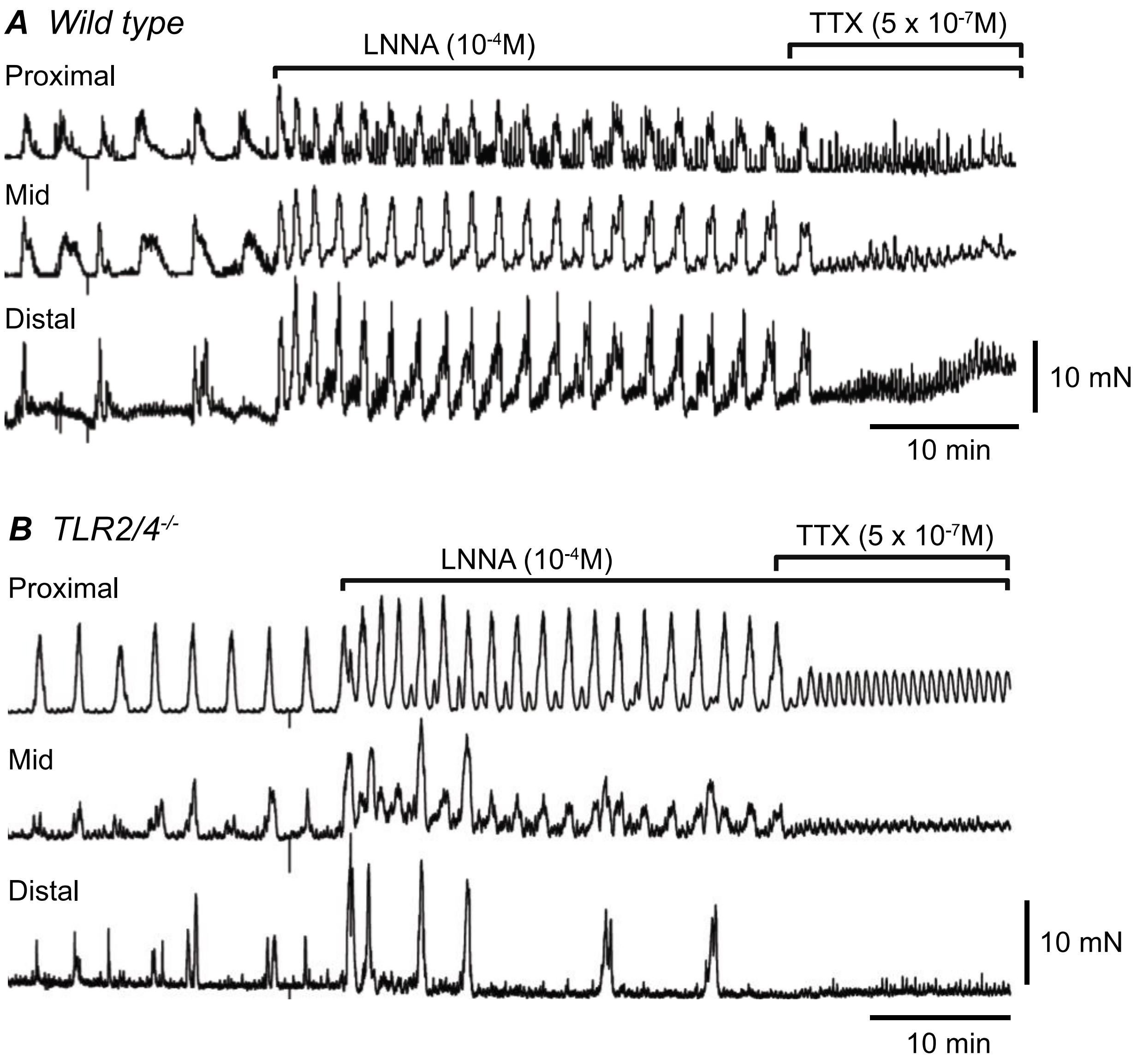


**Supplementary Figure 1.** Example traces illustrating effect of LNNA ($10^{-4}$M) and TTX (5 x $10^{-7}$M) on CMMC activity recorded from proximal, mid and distal sites of WT (A) and *TLR2/4*$^{-/-}$ (B) colon.

**Supplementary Figure 2**

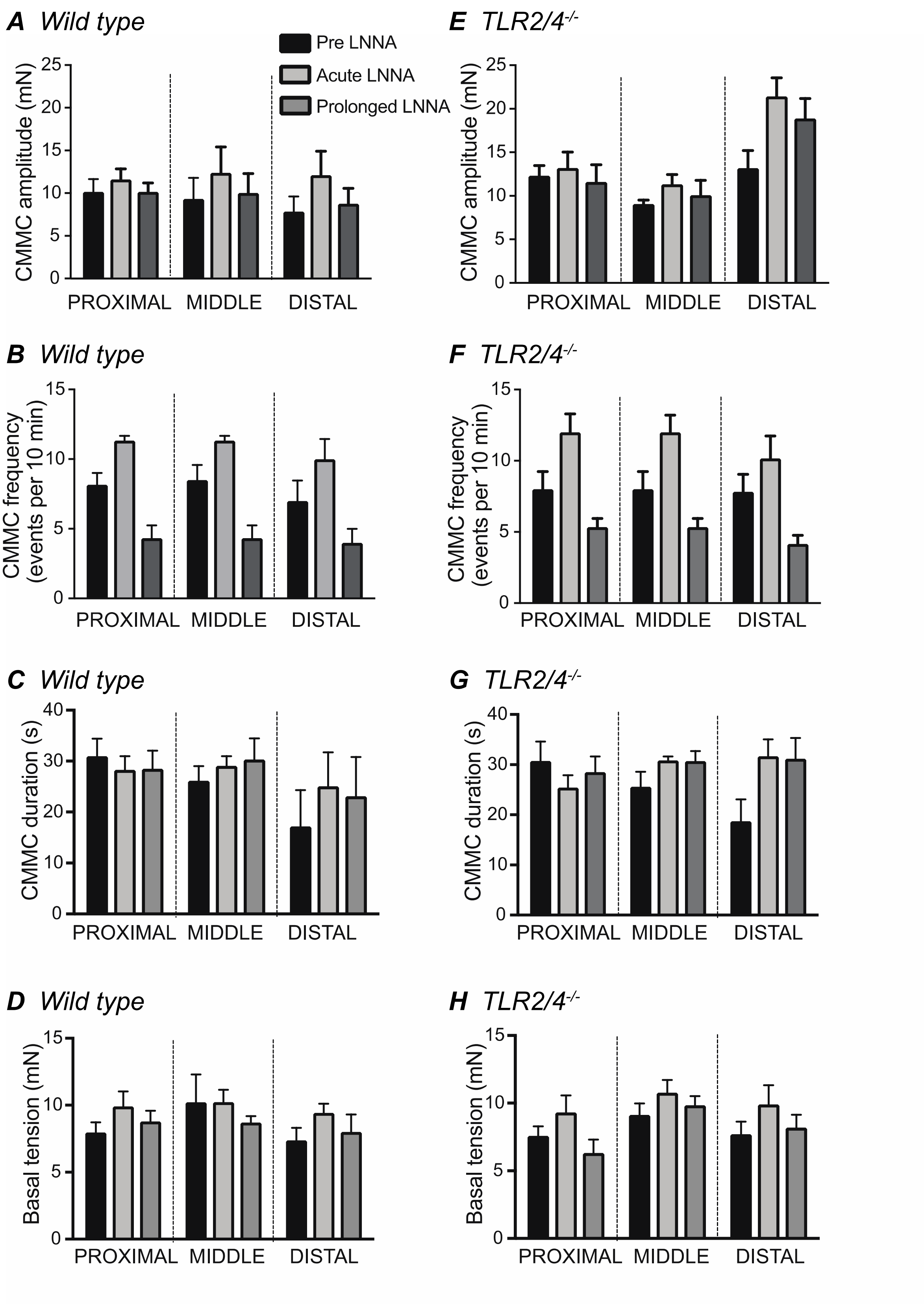


**Supplementary Figure 2.** Summary graphs showing CMMC amplitude (A & E), frequency (B & F), duration (C & G) and basal tone (D & H) prior to the addition of LNNA (pre-LNNA), 5-10 min post application (acute) and 30-40 min post application (prolonged) at proximal, mid and distal sites of WT (A-D) and *TLR2/4*$^{-/-}$ colon.
